# An Introduction to the Proceedings of the *Environmental Release of Engineered Pests: Building an International Governance Framework*

**DOI:** 10.1186/s12919-018-0105-1

**Published:** 2018-07-19

**Authors:** Zachary S. Brown, Lucy Carter, Fred Gould

**Affiliations:** 1 Department of Agricultural and Resource Economics, North Carolina State University, Raleigh, NC USA; 2 Genetic Engineering and Society Center, North Carolina State University, Raleigh, NC USA; 3 Commonwealth Scientific and Industrial Research Organization, Brisbane, Australia; 4 Department of Entomology & Plant Pathology, North Carolina State University, Raleigh, NC USA

## Abstract

In October 2016, a two-day meeting of 65 academic, government and industry professionals was held at North Carolina State University for early-stage discussions about the international governance of *gene drives*: potentially powerful new technologies that can be used for the control of pests, invasive species and disease vectors. Presenters at the meeting prepared seven manuscripts elaborating on the ideas raised. This *BMC Proceedings* issue presents the collection of these peer-reviewed manuscripts.

## What is a gene drive?

Gene drives are products of biotechnology in which a sexually-reproducing organism is genetically engineered with a desirable trait that is passed on to nearly all of its offspring. This contrasts with standard Mendelian inheritance, in which a gene is passed on to approximately half of the organism’s offspring. Working through the mathematical logic of such a system shows that, if a gene drive performs as intended, the desirable trait can be ‘driven’ into entire populations of a species. While this type of approach to controlling evolution has been proposed for decades, recently developed gene editing tools such as CRISPR/Cas9 have dramatically increased the feasibility of practical application [1].

Any trait controlled by one to a few genes could potentially be driven through a sexual population using a gene drive, although there are many characteristics of a population that impact its suitability for gene drive. Currently proposed applications of gene drives fall into two categories: *population replacement* or *suppression*. *Population replacement* drives a genetic modification that adds a desirable trait (or removes an undesirable one). One such application being researched by the group Target Malaria is to genetically modify a species of mosquito that transmits malaria so that it is no longer capable of harboring or transmitting the disease [2]. Similar applications have been investigated for agricultural pests that transmit plant diseases [3]. *Population suppression* drives a deleterious genetic modification that has the capacity to reduce – and possibly eradicate – the species. Such a system may seem paradoxical, since a population suppression gene drive uses evolution to make a species less fit. But researchers have shown that genetic traits such as female sterility can theoretically be driven through a population due to the biased inheritance of gene drives. Once the sterility trait is driven through a sufficient portion of the population, the population should collapse. Proposed applications of population suppression include drives to eliminate or eradicate malaria-transmitting mosquitoes [4]. Another prominent application being developed is a drive for invasive mice on islands that threaten endangered species [5].

## Why a workshop on the international governance of gene drives?

A number of technical challenges still need to be solved to make gene drives feasible [6]. Scientists are also actively researching obvious risks of this technology [7], so as to achieve the seeming paradox of controlling who is impacted, while taking advantage of self-sustaining spread and amplification of these systems. Nevertheless, the broad scope and geographic reach of applications already demand *ex ante* discussion about the ethical and governance challenges. Recent scholarship has significantly advanced this discussion [8, 9]. Yet, with the scope of gene drives’ spread limited by biology, not by national borders, fundamental questions are raised about how deployment of the technology can respect national sovereignty and international norms. These transnational implications also call into question the extent and process for stakeholder engagement. Who is really a stakeholder, when such a technology potentially implicates everyone on Earth?

A number of international treaties may affect global governance of gene drives [10]. For example, the Convention on Biological Diversity (CBD) contains protocols for international liability and redress from transboundary spread of Living Modified Organisms (LMOs), including gene drives. Recent meetings of the CBD’s technical working groups are beginning to address how gene drives will be addressed [11]. The International Plant Protection Convention (IPPC), which currently governs phytosanitary standards, has been applied to international norms surrounding classical biocontrol – another agricultural pest control method that is designed to spread. Additionally, the Codex Alimentarius, used by the World Trade Organization to govern disputes about the international trade of genetically modified organisms (GMOs), could affect agricultural trade in products using gene drives, e.g. for pest control. Finally, the World Health Organization (WHO) has developed guidelines for the use of genetically modified mosquitoes for malaria control [12].

To make sense of these issues and consider if and how an international framework could be developed, this two-day workshop brought together regulators and scientists from a broad cross-section of countries. The first day of the workshop began with presentations from regulators about how agricultural biotechnology is *currently* regulated in their countries, with brief reflections on how something like a gene drive could be governed. On the second half of the first day, academic scholars from the social sciences provided broad socioecological perspectives on how GMO governance and public engagement has unfolded around the world. (The full agenda and presentation slides from the meeting can be found at: https://research.ncsu.edu/ges/research/projects/oecd-crp-meeting/.) Day two continued with additional academic scholars, as well as representatives of private industry, presenting their visions of what an effective global governance framework for gene drives might look like. Workshop participants then transitioned into an interactive, stakeholder mapping exercise [13]: Three case studies for future hypothetical gene drive deployments were posed to the participants. For each of these cases, subgroup participants were asked to identify the potential stakeholders, across national boundaries, in the proposed deployment, and to place each identified stakeholder on a grid according to their perceived influence and interest in the deployment decision. After sharing their findings with the entire workshop group, participants reported that this exercise helped them understand some of the key policy axes to consider in the global governance of gene drives.

## What articles are contained in this BMC proceedings issue?

All workshop presenters agreed in advance to write short manuscripts based around their presentations. We purposefully asked presenters to prepare their manuscripts after the meeting, in order to incorporate lessons learned over the two days of exchange. In the lead article in this issue, Collins [14] sets the stage for discussions of global governance, by summarizing key points from a 2016 report by the U.S. National Academies of Sciences, Engineering and Medicine (NASEM), entitled “Gene drives on the horizon: Advancing science, navigating uncertainty, and aligning research with public values.”

When permitted by their ministries’ rules, the government participants at the workshop produced a number of useful guides on the biotechnology regulatory environments in their jurisdictions. Ahuja ([15], *this issue*) notes that biotechnology regulation in India could in principle encompass gene drive regulation but that rulemaking awaits outcomes at the international level, through the Cartagena Protocol on Biosafety. Glover et al. ([16]*, this issue*) reviews biotechnology regulatory coordination across a number of African countries through the New Partnership for Africa’s Development (NEPAD). Through the African Biosafety Network of Expertise, NEPAD is currently building capacity to coordinate regional regulation of gene drives and other emerging biotechnologies. In Brazil, Andrade et al. ([17]*, this issue*) explains the functioning of the National Biosafety Technical Committee (CTNBio), and how it handles regulation of genetically modified insects. This paper emphasizes the particular importance of stakeholder empowerment in regulatory decision-making, in order to navigate likely “political tensions” arising from these technologies. Regulatory structures for a number of other jurisdictions are described within other articles in this issue.

In a number of cases, the exchange of viewpoints at this workshop resulted in manuscripts being jointly authored by participants with diverse (and sometimes divergent) views on whether gene drives should even be used, let alone how to govern them. The very fact that these participants were able to work together on a common text we consider to be a significant achievement of the meeting.

Turner et al. ([18], *this issue*) use the case of the deliberate release of a genetically modified self-limiting olive fly (*Bactrocera oleae*) in Spain to showcase how existing European Union regulatory and stakeholder engagement frameworks play out in practice. The authors offer a detailed analysis of a process which failed to meet expected outcomes including lessons learned for future applications.

Burgess et al. ([19], *this issue*) explore the significant institutional and operational barriers to designing and implementing processes which engage diverse segments of the public in a manner that builds trust and confidence in science and government.

Stirling et al. ([20], *this issue*) take the discussion further by challenging the fundamental premises and practices of traditional risk assessment processes which often claim to have engaged with the public in some form. The authors make a case for legitimate and inclusive engagement processes to decision-making which better reflect the diversity of a much broader range of stakeholder perspectives and interests.

## Conclusions of the meeting

As one of the earliest public discussions on the international governance challenges of gene drives, the challenges and ideas raised at this meeting helped conceptualize a basis for governance of these technologies. In general, participants widely recognized significant questions about which international conventions should govern gene drives. In 2017, the CBD’s Ad Hoc Technical Expert Group (AHTEG) on Synthetic Biology confirmed that gene drives qualify as LMOs as per the Cartagena Protocol (CBD) [21], indicating the potential jurisdiction of the CBD over the international governance of gene drives. It remains to be seen whether other international conventions or organizations, such as the IPPC or WHO, will attempt to assert any authority over these technologies.

An additional conclusion of the meeting – in particular by Stirling et al. (ibid.) - was that conventional risk assessment methods may be challenged by the unique properties of gene drives. This conclusion was echoed in the 2017 AGHTEG meeting report’s conclusion that “existing risk assessment considerations and methodologies might not be sufficient or adequate to assess and evaluate the risks that might arise from organisms containing engineered gene drives due to limited experience and the complexity of the potential impacts on the environment” [21].

More generally, many meeting participants concluded that gene drives have the potential to significantly alter our global commons. Because of the institutional complexity involved in governing global commons, specialized analytical methods may be useful for identifying opportunities for coordination. One such method is “Institutional Analysis and Development,” introduced by Elinor Ostrom [9]. The stakeholder mapping exercises conducted at the end of the meeting comprise one component of this approach. While these exercises did not necessarily answer the difficult questions posed at the meeting, they did enrich participants’ appreciation for the diversity of stakeholders and values about the outcomes from a potential gene deployment. The exercise also made clear how entrenched the power structures are between the different international stakeholders, and how difficult it is likely to be to empower other stakeholders who have a lot to gain or lose from gene drive releases to influence the trajectory of international governance. One idea discussed for addressing this entrenchment is to layer participatory processes between local, regional and global constituencies for more effective action. Different process layers may allow for more or less one-way or two-way engagement with different stakeholders.

## Who organized and sponsored the workshop?

This meeting was organized by the Genetic Engineering & Society (GES) Center at North Carolina State University and by the Australian-based Commonwealth Scientific and Industrial Research Organization (CSIRO). The meeting was sponsored by the OECD Co-operative Research Programme on Biological Resource Management for Sustainable Agricultural Systems, whose financial support made it possible for most of the invited speakers to participate in the Conference. Additional funding was provided by the North Carolina Biotechnology Center. In-kind support was also provided by the GES Center and CSIRO.
